# (2-Amino-4,6-dimethyl­pyrimidine-κ*N* ^1^)(2-amino-4-methyl­pyrimidine-κ*N* ^1^)silver(I) nitrate

**DOI:** 10.1107/S1600536809035181

**Published:** 2009-09-05

**Authors:** Hua Yang

**Affiliations:** a Department of Chemistry, Mudanjiang Teachers College, Mudanjiang 157012, People’s Republic of China

## Abstract

Colourless crystals of the title compound, [Ag(C_5_H_7_N_3_)(C_6_H_9_N_3_)]NO_3_, separated out of a solution of 2-amino-4-methyl­pyrimidine, 2-amino-4,6-dimethyl­pyrimidine and silver nitrate in water and methanol. The central Ag^I^ ion is coordinated by two different N atoms in the aromatic rings of the ligands, with an N—Ag—N angle of 173.9 (2)°. The crystal structure is composed of two complexed cations and stabilized by an inter­molecular N—H⋯O and N—H⋯N hydrogen-bond network and there is π–π stacking of the aromatic rings [inter­planar distance 3.651 (10) Å].

## Related literature

For N—Ag—N coordination geometries, see: Greenwood & Earnshaw (1997 ▶). For π–π stacking, see: Munakata *et al.* (2000 ▶). For silver coordination networks, see: Seward *et al.* (2004 ▶); Shimizu *et al.* (1999 ▶).
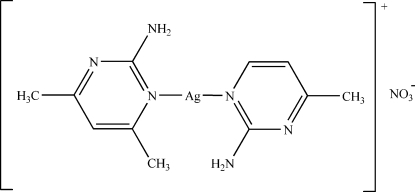


## Experimental

### 

#### Crystal data

[Ag(C_5_H_7_N_3_)(C_6_H_9_N_3_)]NO_3_
*M* *_r_* = 402.18
Orthorhombic, 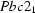

*a* = 7.5689 (4) Å
*b* = 19.1582 (7) Å
*c* = 20.1826 (10) Å
*V* = 2926.6 (2) Å^3^
*Z* = 8
Mo *K*α radiation
μ = 1.40 mm^−1^
*T* = 120 K
0.50 × 0.40 × 0.35 mm

#### Data collection

Bruker APEXII CCD area-detector diffractometer
Absorption correction: multi-scan (*SADABS*; Bruker, 2005 ▶) *T*_min_ = 0.512, *T*_max_ = 0.616
11376 measured reflections
3244 independent reflections
2647 reflections with *I* > 2σ(*I*)
*R*_int_ = 0.027

#### Refinement

*R*[*F* ^2^ > 2σ(*F* ^2^)] = 0.040
*wR*(*F* ^2^) = 0.129
*S* = 1.10
3244 reflections
403 parameters
1 restraint
H-atom parameters constrained
Δρ_max_ = 0.95 e Å^−3^
Δρ_min_ = −0.63 e Å^−3^
Absolute structure: Flack (1983 ▶), Friedel pairs merged
Flack parameter: 0.07 (6)

### 

Data collection: *APEX2* (Bruker, 2005 ▶); cell refinement: *SAINT* (Bruker, 2005 ▶); data reduction: *SAINT*; program(s) used to solve structure: *SHELXS97* (Sheldrick, 2008 ▶); program(s) used to refine structure: *SHELXL97* (Sheldrick, 2008 ▶); molecular graphics: *ORTEP-3 for Windows* (Farrugia, 1997 ▶); software used to prepare material for publication: *WinGX* (Farrugia, 1999 ▶).

## Supplementary Material

Crystal structure: contains datablocks I, global. DOI: 10.1107/S1600536809035181/jh2100sup1.cif

Structure factors: contains datablocks I. DOI: 10.1107/S1600536809035181/jh2100Isup2.hkl

Additional supplementary materials:  crystallographic information; 3D view; checkCIF report

## Figures and Tables

**Table 1:** Hydrogen-bond geometry (Å, °)

*D*—H⋯*A*	*D*—H	H⋯*A*	*D*⋯*A*	*D*—H⋯*A*
N2—H2*B*⋯O5	0.88	2.20	3.004 (10)	152
N2—H2*C*⋯N6^i^	0.88	2.17	3.053 (11)	178
N5—H5*A*⋯N3^ii^	0.88	2.20	3.078 (11)	178
N5—H5*B*⋯O1^ii^	0.88	2.33	3.042 (13)	138
N8—H8*B*⋯O4	0.88	2.36	3.026 (12)	133
N8—H8*C*⋯N12^iii^	0.88	2.20	3.073 (11)	175
N11—H11*A*⋯N9^iv^	0.88	2.15	3.029 (10)	176
N11—H11*B*⋯O2^iv^	0.88	2.16	2.965 (11)	151

Symmetry codes: (i) 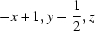; (ii) 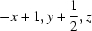; (iii) 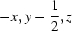; (iv) 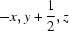.

**Fig. 1.**
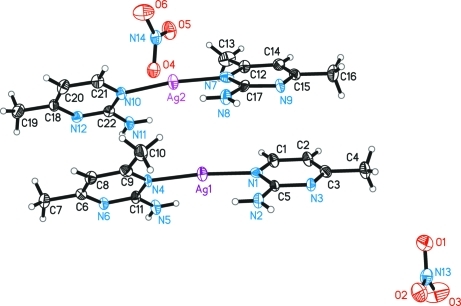
The molecular structure with atom labels and 30% probability displacement ellipsoids for non-H atoms.

**Fig. 2.**
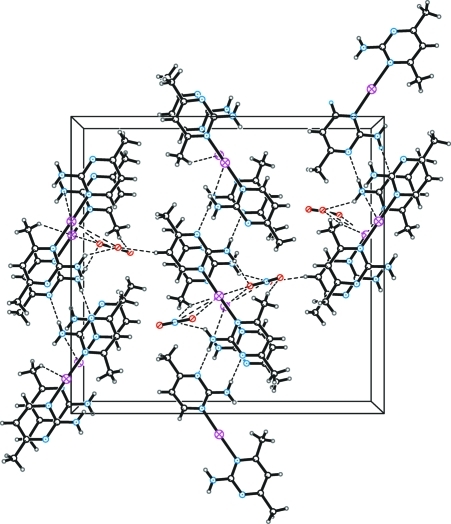
The packing diagram of molecules, viewed down the b axis, with the weak interactions shown as dashed lines.

## supplementary crystallographic information

### Comment

The nitrogen-containing bidentates, heterocyclic pyrimidine compounds are of great interests with capabilities to form stable hydrogen bonding network between aminos and nitrogen atoms of hetero rings. The crystal structure of the title compound (I) comprised of balanced NO_3_^-^ anions and [Ag(2-amino-4-methylpyrimidine)(2-amino-4,6-dimethylpyrimidine)]^+^ cations. The central silver ion, coordinated to two nitrogen atoms from the pyrimidine rings of those two different ligands, giving linear N—Ag—N coordination geometries (Greenwood *et al.*, 1997). The whole crystal structure was stabilized by multiple intermolecular N–H–N hydrogen bonding network and pi-pi stacking with the interplane distance of 3.65 Å (Munakata *et al.*, 2000).

### Experimental

A solution of 108 mg (1 mmol) 2-amino-4-methylpyrimidine and 123 mg (1 mmol) of 2-amino-4,6-dimethylpyrimidine in water-CH_3_OH (1:1 V/V,10 ml) was added to an aqueous solution of AgNO_3_ 170 mg (1 mmol) in 3 ml water with stirring at 333 K. A small amount of white precipitate was removed from the resulting solution. Prism shaped colorless crystals were obtained by slow evaporation of the solvent at room temperature over a period of 3 days.

### Refinement

All H atoms were placed in calculated positions and refined as riding, with C–H = 0.96–0.98 Å, and N–H = 0.88 Å, and *U*_iso_(H) = 1.2 or 1.5*U*_eq_(C,*N*).

### Crystal data

**Table:**

[Ag(C_5_H_7_N_3_)(C_6_H_9_N_3_)]NO_3_	*F*(000) = 1616
*M**_r_* = 402.18	*D*_x_ = 1.826 Mg m^−^^3^
Orthorhombic, *P**b**c*2_1_	Mo *K*α radiation, λ = 0.71073 Å
Hall symbol: P 2c -2b	Cell parameters from 3244 reflections
*a* = 7.5689 (4) Å	θ = 2.9–27.0°
*b* = 19.1582 (7) Å	µ = 1.40 mm^−^^1^
*c* = 20.1826 (10) Å	*T* = 120 K
*V* = 2926.6 (2) Å^3^	Prism, colourless
*Z* = 8	0.50 × 0.40 × 0.35 mm

### Data collection

**Table:**

Bruker APEXII CCD area-detector diffractometer	3244 independent reflections
Radiation source: fine-focus sealed tube	2647 reflections with *I* > 2σ(*I*)
graphite	*R*_int_ = 0.027
φ and ω scans	θ_max_ = 27.0°, θ_min_ = 2.9°
Absorption correction: multi-scan (*SADABS*; Bruker, 2005)	*h* = −8→9
*T*_min_ = 0.512, *T*_max_ = 0.616	*k* = −24→23
11376 measured reflections	*l* = −17→25

### Refinement

**Table:**

Refinement on *F*^2^	Secondary atom site location: difference Fourier map
Least-squares matrix: full	Hydrogen site location: inferred from neighbouring sites
*R*[*F*^2^ > 2σ(*F*^2^)] = 0.040	H-atom parameters constrained
*wR*(*F*^2^) = 0.129	*w* = 1/[σ^2^(*F*_o_^2^) + (0.0822*P*)^2^ + 2.1516*P*] where *P* = (*F*_o_^2^ + 2*F*_c_^2^)/3
*S* = 1.10	(Δ/σ)_max_ = 0.010
3244 reflections	Δρ_max_ = 0.95 e Å^−^^3^
403 parameters	Δρ_min_ = −0.63 e Å^−^^3^
1 restraint	Absolute structure: Flack (1983), 0 Friedel pairs
Primary atom site location: structure-invariant direct methods	Flack parameter: 0.07 (6)

### Special details

**Table:**

Geometry. All e.s.d.'s (except the e.s.d. in the dihedral angle between two l.s. planes) are estimated using the full covariance matrix. The cell e.s.d.'s are taken into account individually in the estimation of e.s.d.'s in distances, angles and torsion angles; correlations between e.s.d.'s in cell parameters are only used when they are defined by crystal symmetry. An approximate (isotropic) treatment of cell e.s.d.'s is used for estimating e.s.d.'s involving l.s. planes.
Refinement. Refinement of *F*^2^ against ALL reflections. The weighted *R*-factor *wR* and goodness of fit *S* are based on *F*^2^, conventional *R*-factors *R* are based on *F*, with *F* set to zero for negative *F*^2^. The threshold expression of *F*^2^ > σ(*F*^2^) is used only for calculating *R*-factors(gt) *etc*. and is not relevant to the choice of reflections for refinement. *R*-factors based on *F*^2^ are statistically about twice as large as those based on *F*, and *R*- factors based on ALL data will be even larger.

### Fractional atomic coordinates and isotropic or equivalent isotropic displacement parameters (Å^2^)

**Table:**

	*x*	*y*	*z*	*U*_iso_*/*U*_eq_	
Ag1	0.71751 (9)	0.60684 (3)	0.47181 (3)	0.0436 (2)	
Ag2	0.21246 (9)	0.64903 (3)	0.49169 (4)	0.0463 (2)	
C1	0.7635 (13)	0.5144 (5)	0.3524 (6)	0.045 (2)	
H1A	0.8144	0.5561	0.3356	0.054*	
C2	0.7561 (14)	0.4570 (5)	0.3120 (6)	0.046 (2)	
H2A	0.7993	0.4585	0.2678	0.055*	
C3	0.6815 (11)	0.3954 (5)	0.3386 (5)	0.040 (2)	
C4	0.6715 (17)	0.3304 (6)	0.2971 (6)	0.058 (3)	
H4A	0.6587	0.2896	0.3261	0.087*	
H4B	0.7798	0.3258	0.2709	0.087*	
H4C	0.5694	0.3333	0.2674	0.087*	
C5	0.6273 (11)	0.4538 (4)	0.4376 (5)	0.0337 (19)	
C6	0.6599 (11)	0.8125 (4)	0.6122 (5)	0.0352 (17)	
C7	0.6426 (15)	0.8776 (5)	0.6539 (6)	0.053 (2)	
H7A	0.5347	0.9026	0.6415	0.080*	
H7B	0.7453	0.9078	0.6466	0.080*	
H7C	0.6366	0.8646	0.7008	0.080*	
C8	0.7394 (12)	0.7544 (5)	0.6393 (5)	0.0381 (19)	
H8A	0.7776	0.7534	0.6841	0.046*	
C9	0.7610 (12)	0.6963 (5)	0.5967 (5)	0.0373 (19)	
C10	0.8413 (15)	0.6312 (6)	0.6203 (7)	0.058 (3)	
H10A	0.9142	0.6110	0.5850	0.087*	
H10B	0.7481	0.5981	0.6325	0.087*	
H10C	0.9153	0.6409	0.6590	0.087*	
C11	0.6221 (11)	0.7588 (4)	0.5118 (4)	0.0349 (18)	
C12	0.2777 (12)	0.5562 (5)	0.3701 (5)	0.0367 (19)	
C13	0.3473 (17)	0.6232 (6)	0.3442 (7)	0.061 (3)	
H13A	0.4342	0.6422	0.3754	0.091*	
H13B	0.2500	0.6565	0.3390	0.091*	
H13C	0.4039	0.6153	0.3012	0.091*	
C14	0.2742 (12)	0.4978 (6)	0.3302 (5)	0.041 (2)	
H14A	0.3211	0.4978	0.2866	0.050*	
C15	0.1979 (10)	0.4393 (5)	0.3581 (5)	0.0377 (19)	
C16	0.1861 (17)	0.3734 (6)	0.3177 (6)	0.060 (3)	
H16A	0.2005	0.3328	0.3469	0.090*	
H16B	0.2797	0.3733	0.2841	0.090*	
H16C	0.0706	0.3711	0.2959	0.090*	
C17	0.1461 (11)	0.4950 (4)	0.4551 (4)	0.0317 (17)	
C18	0.1404 (11)	0.8570 (4)	0.6286 (4)	0.0330 (16)	
C19	0.1264 (14)	0.9230 (5)	0.6686 (6)	0.055 (3)	
H19A	0.0081	0.9430	0.6631	0.082*	
H19B	0.2150	0.9566	0.6531	0.082*	
H19C	0.1468	0.9125	0.7154	0.082*	
C20	0.2179 (12)	0.7976 (5)	0.6557 (5)	0.042 (2)	
H20A	0.2582	0.7960	0.7002	0.050*	
C21	0.2322 (12)	0.7409 (5)	0.6130 (6)	0.039 (2)	
H21A	0.2862	0.6996	0.6294	0.046*	
C22	0.0990 (11)	0.8014 (5)	0.5295 (4)	0.0325 (18)	
N1	0.7026 (8)	0.5141 (4)	0.4139 (4)	0.0329 (15)	
N2	0.5607 (11)	0.4498 (4)	0.4978 (4)	0.0439 (18)	
H2B	0.5637	0.4865	0.5241	0.053*	
H2C	0.5133	0.4105	0.5117	0.053*	
N3	0.6194 (9)	0.3939 (3)	0.3996 (4)	0.0372 (16)	
N4	0.7038 (9)	0.6994 (3)	0.5340 (4)	0.0303 (15)	
N5	0.5602 (11)	0.7605 (4)	0.4515 (4)	0.0441 (19)	
H5A	0.5064	0.7982	0.4369	0.053*	
H5B	0.5722	0.7240	0.4255	0.053*	
N6	0.6023 (9)	0.8151 (3)	0.5505 (4)	0.0353 (15)	
N7	0.2130 (9)	0.5570 (4)	0.4326 (4)	0.0378 (18)	
N8	0.0846 (11)	0.4947 (4)	0.5176 (4)	0.0446 (19)	
H8B	0.0888	0.5331	0.5416	0.054*	
H8C	0.0402	0.4562	0.5345	0.054*	
N9	0.1322 (10)	0.4362 (3)	0.4176 (4)	0.0351 (15)	
N10	0.1754 (9)	0.7407 (3)	0.5510 (4)	0.0351 (16)	
N11	0.0341 (10)	0.8033 (3)	0.4697 (4)	0.0407 (16)	
H11A	−0.0189	0.8413	0.4553	0.049*	
H11B	0.0434	0.7666	0.4437	0.049*	
N12	0.0822 (9)	0.8582 (3)	0.5674 (4)	0.0349 (15)	
N13	0.1299 (12)	0.1949 (4)	0.3307 (4)	0.0475 (19)	
N14	0.3858 (11)	0.5590 (4)	0.6325 (4)	0.0424 (17)	
O1	0.2935 (10)	0.1966 (5)	0.3249 (6)	0.076 (3)	
O2	0.0732 (12)	0.1825 (4)	0.3870 (4)	0.067 (2)	
O3	0.0361 (15)	0.2039 (8)	0.2829 (6)	0.114 (4)	
O4	0.2254 (10)	0.5607 (5)	0.6432 (4)	0.059 (2)	
O5	0.4378 (11)	0.5740 (4)	0.5762 (4)	0.0595 (19)	
O6	0.4910 (11)	0.5443 (5)	0.6767 (6)	0.076 (3)	

### Atomic displacement parameters (Å^2^)

**Table:**

	*U*^11^	*U*^22^	*U*^33^	*U*^12^	*U*^13^	*U*^23^
Ag1	0.0552 (4)	0.0293 (3)	0.0463 (4)	0.0003 (2)	0.0008 (5)	−0.0078 (3)
Ag2	0.0636 (4)	0.0301 (3)	0.0451 (4)	0.0007 (3)	0.0003 (5)	−0.0077 (3)
C1	0.053 (5)	0.033 (5)	0.048 (6)	0.001 (4)	0.014 (5)	0.006 (5)
C2	0.059 (6)	0.039 (5)	0.041 (6)	0.005 (4)	0.006 (4)	−0.003 (4)
C3	0.036 (4)	0.038 (4)	0.047 (5)	0.006 (3)	0.000 (4)	−0.004 (4)
C4	0.073 (7)	0.046 (5)	0.055 (7)	−0.001 (5)	0.004 (5)	−0.018 (5)
C5	0.032 (4)	0.028 (4)	0.041 (5)	0.007 (3)	−0.006 (4)	−0.014 (4)
C6	0.037 (4)	0.038 (4)	0.030 (4)	−0.002 (4)	0.004 (3)	−0.009 (3)
C7	0.056 (5)	0.051 (5)	0.053 (6)	−0.001 (5)	−0.008 (5)	−0.022 (5)
C8	0.044 (4)	0.032 (4)	0.038 (5)	0.003 (4)	−0.002 (4)	−0.002 (4)
C9	0.040 (4)	0.036 (5)	0.036 (5)	−0.005 (4)	0.011 (4)	0.001 (4)
C10	0.058 (6)	0.064 (6)	0.053 (7)	−0.001 (5)	0.003 (5)	−0.007 (6)
C11	0.047 (5)	0.028 (4)	0.029 (4)	−0.011 (3)	0.010 (4)	−0.006 (3)
C12	0.047 (5)	0.038 (5)	0.025 (4)	0.000 (4)	0.001 (4)	0.003 (4)
C13	0.070 (7)	0.054 (6)	0.058 (8)	−0.011 (6)	−0.001 (6)	0.018 (6)
C14	0.041 (4)	0.055 (6)	0.028 (5)	0.012 (4)	−0.013 (4)	−0.005 (4)
C15	0.037 (4)	0.038 (4)	0.038 (5)	0.007 (3)	−0.003 (4)	−0.008 (4)
C16	0.082 (8)	0.047 (5)	0.052 (7)	0.003 (5)	−0.004 (6)	−0.014 (5)
C17	0.037 (4)	0.033 (4)	0.025 (4)	−0.002 (3)	−0.010 (3)	0.001 (3)
C18	0.041 (4)	0.026 (4)	0.032 (4)	−0.004 (3)	0.002 (3)	−0.008 (3)
C19	0.060 (6)	0.043 (5)	0.060 (7)	0.002 (4)	−0.017 (5)	−0.015 (5)
C20	0.056 (5)	0.041 (5)	0.029 (4)	−0.011 (4)	−0.003 (4)	−0.004 (4)
C21	0.048 (5)	0.035 (4)	0.033 (5)	−0.001 (4)	0.003 (4)	0.002 (4)
C22	0.033 (4)	0.033 (4)	0.031 (5)	−0.011 (3)	0.010 (3)	0.002 (3)
N1	0.030 (3)	0.026 (3)	0.043 (4)	0.007 (2)	−0.001 (3)	0.002 (3)
N2	0.056 (4)	0.036 (3)	0.040 (4)	−0.004 (3)	0.004 (4)	−0.013 (4)
N3	0.042 (4)	0.027 (3)	0.042 (4)	0.002 (3)	−0.002 (3)	−0.012 (3)
N4	0.041 (4)	0.018 (3)	0.031 (4)	−0.002 (2)	0.005 (3)	−0.005 (3)
N5	0.065 (5)	0.037 (4)	0.031 (4)	0.008 (4)	0.000 (4)	−0.006 (3)
N6	0.039 (3)	0.027 (3)	0.040 (4)	0.004 (3)	0.001 (3)	−0.006 (3)
N7	0.034 (3)	0.040 (4)	0.040 (5)	0.004 (3)	−0.006 (3)	0.015 (3)
N8	0.062 (5)	0.035 (4)	0.036 (4)	−0.008 (3)	0.008 (4)	−0.007 (3)
N9	0.051 (4)	0.027 (3)	0.027 (4)	0.008 (3)	−0.008 (3)	−0.003 (3)
N10	0.042 (4)	0.021 (3)	0.043 (4)	−0.001 (3)	0.001 (3)	0.001 (3)
N11	0.056 (4)	0.030 (3)	0.036 (4)	0.008 (3)	−0.004 (4)	−0.001 (3)
N12	0.031 (3)	0.031 (3)	0.043 (4)	−0.002 (3)	−0.002 (3)	−0.001 (3)
N13	0.064 (5)	0.046 (4)	0.033 (4)	−0.001 (4)	−0.008 (4)	−0.004 (3)
N14	0.057 (5)	0.042 (4)	0.028 (4)	0.002 (3)	−0.001 (3)	−0.006 (3)
O1	0.056 (5)	0.078 (6)	0.092 (8)	0.006 (4)	−0.004 (5)	−0.023 (6)
O2	0.081 (5)	0.061 (5)	0.058 (5)	0.012 (4)	−0.002 (4)	−0.007 (4)
O3	0.089 (6)	0.190 (12)	0.061 (7)	−0.021 (8)	−0.027 (6)	0.035 (8)
O4	0.057 (4)	0.082 (6)	0.037 (4)	0.009 (4)	−0.005 (3)	−0.014 (4)
O5	0.080 (5)	0.056 (4)	0.042 (4)	−0.003 (4)	0.014 (4)	−0.002 (3)
O6	0.064 (5)	0.088 (6)	0.074 (7)	0.010 (4)	−0.007 (5)	0.018 (5)

### Geometric parameters (Å, °)

**Table:**

Ag1—N1	2.130 (8)	C13—H13C	0.9800
Ag1—N4	2.176 (6)	C14—C15	1.380 (15)
Ag2—N10	2.145 (7)	C14—H14A	0.9500
Ag2—N7	2.129 (9)	C15—N9	1.302 (12)
C1—N1	1.324 (14)	C15—C16	1.506 (14)
C1—C2	1.371 (16)	C16—H16A	0.9800
C1—H1A	0.9500	C16—H16B	0.9800
C2—C3	1.415 (14)	C16—H16C	0.9800
C2—H2A	0.9500	C17—N8	1.345 (11)
C3—N3	1.316 (13)	C17—N7	1.368 (11)
C3—C4	1.503 (14)	C17—N9	1.361 (11)
C4—H4A	0.9800	C18—N12	1.313 (11)
C4—H4B	0.9800	C18—C20	1.391 (13)
C4—H4C	0.9800	C18—C19	1.503 (12)
C5—N2	1.317 (13)	C19—H19A	0.9800
C5—N1	1.374 (12)	C19—H19B	0.9800
C5—N3	1.382 (11)	C19—H19C	0.9800
C6—N6	1.320 (12)	C20—C21	1.391 (14)
C6—C8	1.378 (13)	C20—H20A	0.9500
C6—C7	1.511 (13)	C21—N10	1.322 (14)
C7—H7A	0.9800	C21—H21A	0.9500
C7—H7B	0.9800	C22—N11	1.304 (12)
C7—H7C	0.9800	C22—N12	1.336 (11)
C8—C9	1.416 (14)	C22—N10	1.369 (11)
C8—H8A	0.9500	N2—H2B	0.8800
C9—N4	1.338 (14)	N2—H2C	0.8800
C9—C10	1.467 (15)	N5—H5A	0.8800
C10—H10A	0.9800	N5—H5B	0.8800
C10—H10B	0.9800	N8—H8B	0.8800
C10—H10C	0.9800	N8—H8C	0.8800
C11—N5	1.304 (11)	N11—H11A	0.8800
C11—N6	1.340 (11)	N11—H11B	0.8800
C11—N4	1.370 (11)	N13—O3	1.210 (13)
C12—N7	1.353 (13)	N13—O1	1.244 (12)
C12—C14	1.379 (14)	N13—O2	1.238 (12)
C12—C13	1.482 (13)	N14—O5	1.237 (11)
C13—H13A	0.9800	N14—O4	1.233 (11)
C13—H13B	0.9800	N14—O6	1.229 (12)
			
N1—Ag1—N4	173.9 (2)	C15—C16—H16B	109.5
N10—Ag2—N7	172.6 (3)	H16A—C16—H16B	109.5
N1—C1—C2	122.7 (10)	C15—C16—H16C	109.5
N1—C1—H1A	118.6	H16A—C16—H16C	109.5
C2—C1—H1A	118.6	H16B—C16—H16C	109.5
C1—C2—C3	117.3 (10)	N8—C17—N7	116.4 (8)
C1—C2—H2A	121.4	N8—C17—N9	119.4 (8)
C3—C2—H2A	121.4	N7—C17—N9	124.2 (8)
N3—C3—C2	121.1 (9)	N12—C18—C20	121.7 (8)
N3—C3—C4	119.0 (9)	N12—C18—C19	117.8 (8)
C2—C3—C4	119.9 (10)	C20—C18—C19	120.5 (8)
C3—C4—H4A	109.5	C18—C19—H19A	109.5
C3—C4—H4B	109.5	C18—C19—H19B	109.5
H4A—C4—H4B	109.5	H19A—C19—H19B	109.5
C3—C4—H4C	109.5	C18—C19—H19C	109.5
H4A—C4—H4C	109.5	H19A—C19—H19C	109.5
H4B—C4—H4C	109.5	H19B—C19—H19C	109.5
N2—C5—N1	121.9 (8)	C21—C20—C18	115.4 (9)
N2—C5—N3	116.6 (8)	C21—C20—H20A	122.3
N1—C5—N3	121.5 (8)	C18—C20—H20A	122.3
N6—C6—C8	123.3 (9)	C20—C21—N10	124.2 (9)
N6—C6—C7	117.8 (8)	C20—C21—H21A	117.9
C8—C6—C7	118.9 (9)	N10—C21—H21A	117.9
C6—C7—H7A	109.5	N11—C22—N12	118.1 (8)
C6—C7—H7B	109.5	N11—C22—N10	118.5 (8)
H7A—C7—H7B	109.5	N12—C22—N10	123.4 (8)
C6—C7—H7C	109.5	C1—N1—C5	118.3 (8)
H7A—C7—H7C	109.5	C1—N1—Ag1	119.5 (7)
H7B—C7—H7C	109.5	C5—N1—Ag1	122.2 (6)
C6—C8—C9	116.4 (9)	C5—N2—H2B	120.0
C6—C8—H8A	121.8	C5—N2—H2C	120.0
C9—C8—H8A	121.8	H2B—N2—H2C	120.0
N4—C9—C8	120.0 (9)	C3—N3—C5	119.0 (8)
N4—C9—C10	118.6 (9)	C9—N4—C11	119.6 (7)
C8—C9—C10	121.3 (10)	C9—N4—Ag1	119.5 (6)
C9—C10—H10A	109.5	C11—N4—Ag1	120.6 (6)
C9—C10—H10B	109.5	C11—N5—H5A	120.0
H10A—C10—H10B	109.5	C11—N5—H5B	120.0
C9—C10—H10C	109.5	H5A—N5—H5B	120.0
H10A—C10—H10C	109.5	C6—N6—C11	118.9 (8)
H10B—C10—H10C	109.5	C12—N7—C17	115.8 (8)
N5—C11—N6	118.9 (9)	C12—N7—Ag2	122.1 (6)
N5—C11—N4	119.3 (8)	C17—N7—Ag2	122.1 (6)
N6—C11—N4	121.8 (8)	C17—N8—H8B	120.0
N7—C12—C14	123.0 (9)	C17—N8—H8C	120.0
N7—C12—C13	116.6 (9)	H8B—N8—H8C	120.0
C14—C12—C13	120.3 (10)	C15—N9—C17	116.5 (8)
C12—C13—H13A	109.5	C21—N10—C22	115.8 (8)
C12—C13—H13B	109.5	C21—N10—Ag2	119.2 (6)
H13A—C13—H13B	109.5	C22—N10—Ag2	125.0 (6)
C12—C13—H13C	109.5	C22—N11—H11A	120.0
H13A—C13—H13C	109.5	C22—N11—H11B	120.0
H13B—C13—H13C	109.5	H11A—N11—H11B	120.0
C15—C14—C12	115.5 (9)	C22—N12—C18	119.5 (7)
C15—C14—H14A	122.3	O3—N13—O1	120.4 (11)
C12—C14—H14A	122.3	O3—N13—O2	123.8 (10)
N9—C15—C14	124.9 (9)	O1—N13—O2	115.8 (10)
N9—C15—C16	116.0 (9)	O5—N14—O4	117.9 (8)
C14—C15—C16	119.1 (9)	O5—N14—O6	120.9 (9)
C15—C16—H16A	109.5	O4—N14—O6	121.1 (9)
			
N1—C1—C2—C3	−0.8 (16)	N6—C11—N4—Ag1	175.8 (6)
C1—C2—C3—N3	1.0 (15)	N1—Ag1—N4—C9	95 (3)
C1—C2—C3—C4	−179.2 (10)	N1—Ag1—N4—C11	−79 (3)
N6—C6—C8—C9	1.4 (13)	C8—C6—N6—C11	−0.4 (13)
C7—C6—C8—C9	−176.5 (8)	C7—C6—N6—C11	177.6 (8)
C6—C8—C9—N4	−0.7 (13)	N5—C11—N6—C6	177.8 (8)
C6—C8—C9—C10	−179.0 (9)	N4—C11—N6—C6	−1.4 (12)
N7—C12—C14—C15	0.6 (13)	C14—C12—N7—C17	1.5 (12)
C13—C12—C14—C15	−176.6 (9)	C13—C12—N7—C17	178.8 (9)
C12—C14—C15—N9	−0.2 (13)	C14—C12—N7—Ag2	−177.9 (7)
C12—C14—C15—C16	179.2 (8)	C13—C12—N7—Ag2	−0.6 (11)
N12—C18—C20—C21	1.3 (13)	N8—C17—N7—C12	178.7 (8)
C19—C18—C20—C21	−176.7 (9)	N9—C17—N7—C12	−4.2 (12)
C18—C20—C21—N10	−1.1 (14)	N8—C17—N7—Ag2	−2.0 (10)
C2—C1—N1—C5	−0.7 (14)	N9—C17—N7—Ag2	175.2 (6)
C2—C1—N1—Ag1	−179.1 (8)	N10—Ag2—N7—C12	117 (2)
N2—C5—N1—C1	−178.7 (9)	N10—Ag2—N7—C17	−63 (3)
N3—C5—N1—C1	2.0 (12)	C14—C15—N9—C17	−2.2 (12)
N2—C5—N1—Ag1	−0.4 (11)	C16—C15—N9—C17	178.4 (8)
N3—C5—N1—Ag1	−179.7 (6)	N8—C17—N9—C15	−178.4 (8)
N4—Ag1—N1—C1	134 (3)	N7—C17—N9—C15	4.5 (12)
N4—Ag1—N1—C5	−44 (3)	C20—C21—N10—C22	0.2 (13)
C2—C3—N3—C5	0.2 (13)	C20—C21—N10—Ag2	178.9 (7)
C4—C3—N3—C5	−179.5 (9)	N11—C22—N10—C21	−177.3 (8)
N2—C5—N3—C3	178.9 (8)	N12—C22—N10—C21	0.5 (12)
N1—C5—N3—C3	−1.8 (12)	N11—C22—N10—Ag2	4.1 (10)
C8—C9—N4—C11	−0.9 (12)	N12—C22—N10—Ag2	−178.2 (5)
C10—C9—N4—C11	177.5 (8)	N7—Ag2—N10—C21	115 (2)
C8—C9—N4—Ag1	−174.8 (6)	N7—Ag2—N10—C22	−66 (3)
C10—C9—N4—Ag1	3.6 (11)	N11—C22—N12—C18	177.5 (8)
N5—C11—N4—C9	−177.2 (8)	N10—C22—N12—C18	−0.2 (12)
N6—C11—N4—C9	2.0 (12)	C20—C18—N12—C22	−0.7 (12)
N5—C11—N4—Ag1	−3.4 (11)	C19—C18—N12—C22	177.3 (8)

### Hydrogen-bond geometry (Å, °)

**Table:**

*D*—H···*A*	*D*—H	H···*A*	*D*···*A*	*D*—H···*A*
N2—H2B···O5	0.88	2.20	3.004 (10)	152
N2—H2C···N6^i^	0.88	2.17	3.053 (11)	178
N5—H5A···N3^ii^	0.88	2.20	3.078 (11)	178
N5—H5B···O1^ii^	0.88	2.33	3.042 (13)	138
N8—H8B···O4	0.88	2.36	3.026 (12)	133
N8—H8C···N12^iii^	0.88	2.20	3.073 (11)	175
N11—H11A···N9^iv^	0.88	2.15	3.029 (10)	176
N11—H11B···O2^iv^	0.88	2.16	2.965 (11)	151

Symmetry codes: (i) −*x*+1, *y*−1/2, *z*; (ii) −*x*+1, *y*+1/2, *z*; (iii) −*x*, *y*−1/2, *z*; (iv) −*x*, *y*+1/2, *z*.
